# Tracking internet interest in anabolic-androgenic steroids using Google Trends

**DOI:** 10.1016/j.drugpo.2017.11.001

**Published:** 2018-01

**Authors:** Joseph Tay Wee Teck, Mark McCann

**Affiliations:** MRC/CSO Social and Public Health Sciences Unit, University of Glasgow, United Kingdom

## Introduction

Anabolic-androgenic steroid (AAS) use, primarily by young men intent on increasing muscularity, is thought to be rising in prevalence (Evans-Brown, McVeigh, Perkins, & Bellis, 2012). This non-medical use of AAS is associated with significant cardiovascular, endocrine, psychological and psychosocial morbidity (Nieschlag and Vorona, 2015a, Nieschlag and Vorona, 2015b). AAS are frequently injected and users now form the largest group using Needle and Syringe Programmes (NSP) in the UK (Hope et al., 2016). The public health significance of AAS injecting becomes more significant with the identification of rising rates of blood borne virus prevalence in this group (Hope et al., 2016).

Despite the potential implications of AAS use both to the individual and the larger population, there is an acknowledged lack of real-time prevalence data (Antonopoulos & Hall, 2016). The best available data come from the Crime Survey for England and Wales, showing that in 2014/15 293,000 people used AAS in their lifetime with 73,000 having used them in the last 6 months (Home Office, 2015). The reliance of self-reporting of AAS use from a secretive group, uncertainty among users about the consumed substances and indeed what is defined as AAS limits the usefulness of this data (Home Office, 2015). Increasing numbers of AAS users presenting to NSP, alongside arrests, drug seizures, media reports, internet forum monitoring and case reports provide a picture of increasing prevalence (Evans-Brown et al., 2012). Nevertheless, current data sources do not appear to provide a real-time indication of variation in prevalence, potentially limiting the responsiveness of services and public health policy.

The Internet has been identified as a key aspect for facilitating AAS use (Brennan, Wells, & Van Hout, 2016), providing ready access to supply (Cordaro, Lombardo, & Cosentino, 2011), information (Andreasson and Johansson, 2015, Brennan et al., 2016) and community (Andreasson & Johansson, 2015). Furthermore, it is increasingly identified as a key driver for the dissemination of media representations of physical ideals, with consistent evidence associating social media usage with body dissatisfaction (Sassatelli, 2010).

Internet searches, for example through Google, are used to access information and consumer products online These searches provide data streams which can then be analysed through online services such as Google Trends (GT). GT is a free, open access online portal which allows users to analyse part of 3.5 billion daily Google searches (Google, 2016). This internet tool provides data on geographical and temporal patterns in user-specified search terms. Epidemiologists are increasingly cognisant of the value of this data to complement traditional sources, providing actionable intelligence to policy-makers (Nuti et al., 2014). Examples of GT research includes the tracking of flu epidemics (Eysenbach, 2011), and the use of drugs such as novel psychoactive substances (Deluca et al., 2012) and synthetic cannabinoids (Curtis et al., 2015). The latter two examples highlight the use of GT in situations where traditional systems may be lacking and where the Internet plays an important role in consumption. Consequently, this study is intended to assess the feasibility of using GT to supplement what we know about AAS related behaviour.

## Methods

This study was approved by the University of Glasgow College of Social Sciences ethics committee. Reference was made to Nuti et al.’s (2014) guidelines for robust GT research. An important issue in performing the analysis was to identify search terms which corresponded closely with what was available for sale online. Cordaro et al. (2011) identified 15 AAS compounds available for sale through the internet. This research, while being 8 years old, nevertheless corresponds with recent AAS user surveys of commonly used compounds (Bates & McVeigh, 2016).

Where an AAS was known by a generic and brand name, the term that produced the greater search volume was chosen. This approach has its limitations; in particular as systematic variations may exist depending on whether a generic or brand name was used. Combining the searches was avoided in this study due to the risk of double counting as the charts for generic and brand names often produced similar time trends. When performing a GT query, the user interface allows for the narrowing down of the scope of the search to set categories such as business, health or sports. All categories were allowed in the search as non-medical AAS use may be viewed in different ways, for example as a health issue or part of fitness and beauty consumerism. The GT data were accessed and downloaded on 14 January 2016. The time period selected was from 1 January 2011 to 31 December 2015. This start date was selected as the GT interface reported an improvement in geo-locating data at this time. A significant limitation of GT for research is that Google maintains a high level of secrecy around their algorithms (Eysenbach, 2011), and more details on this improvement is unavailable. The GT searches were restricted to the UK as this was the area of interest for this project. It is important to note that a worldwide trend analysis would be difficult to interpret, due to differences in legislation and socio-cultural variations in AAS use.

The individual AAS names were entered systematically into the GT interface. This interface allows for a simultaneous comparison of 5 search terms. The least popular search term was removed each time such that the top five were identified. The process was repeated in a second GT page and the next five, less popular search terms were identified. This process was necessary as all data points in each time series are normalised by GT such that they are relative to the highest data point designated 100 (Google, 2016). The data points produced represent the relative search volume (RSV), defined as the total number of searches for that term divided by the total number of Google searches for the specified location and time (Google, 2016). Of the 15 AAS identified by Cordaro et al. (2011), 10 provided sufficient data to produce a time-trend chart. 5 of the steroid compounds did not generate sufficient data points for comparative time-trend analysis. The data points were downloaded as .csv files for further analysis.

## Statistical analysis

The R statistical package with the DeducerHansel Graphical User Interface (GUI) package version 0.4 (Hacker, 2013) was used to run multiplicative seasonal decomposition, which allowed for visual inspection of the components of the time series. Visual inspection was used to determine months of upward and downward inflection for subsequent statistical tests. The Wilcoxon signed-rank test was applied to determine if there were significant differences between search volumes in the peak (April-July) and trough (September-December) months identified from the time series decomposition. Both approaches are commonly used in assessing seasonality in time series analysis (Nwogu, Iwueze & Nlebedim, 2016). The Mann-Kendall test was used to detect overall trends significantly larger than the variance in the data for each search term. We modified significance points using the Bonferroni correction to adjust for multiple testing. All *p*-values and tests are reported.

## Results

**Fig. 1:**
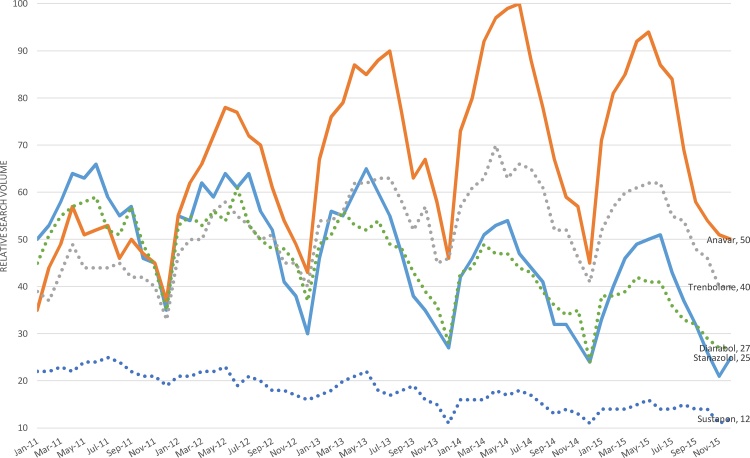
Google Trends relative search volume for more popular AAS in the UK (Jan 2011-Dec 2015).

Table 1 lists the individual AAS ranked in order of popularity based on RSV and the results of statistical testing. The individual decomposition graphs for each AAS is available in the Supplementary material. Fig. 1 reproduces the GT output for the 5 more popular AAS with the output for the less popular AAS in the Supplementary material. A seasonal component for each AAS was identified with peaks occurring in April to July and troughs from September to December. This seasonality was statistically significant for all the AAS except Testosterone enanthate when tested with the Wilcoxon signed-rank test.

**Table 1:** Statistical tests for Google Trends data.

AAS	Relative search volume	Seasonality present on decomposition	Wilcoxon signed rank test (Sep-Dec/Apr-Jul)	Seasonal Mann Kendall Test
		More popular		
Anavar	67	Y	p < 0.0001**	p < 0.0001** Tau = 0.628
Trenbolone	52	Y	p < 0.0001**	p < 0.0001** Tau = 0.554
Stanozolol	47	Y	p < 0.0001**	p < 0.0001** Tau = −0.778
Dianabol	45	Y	P < 0.0001**	P < 0.0001** Tau = −0.829
Sustanon	23	Y	P < 0.0001**	p < 0.0001** Tau = −0.907
		Less popular		
Nandralone	11	Y	p < 0.0001**	p < 0.0001** Tau = −0.756
Boldenone	9	Y	P < 0.000***	p = 0.254 Tau = 0.144
Masteron	9	Y	p < 0.0001**	P < 0.002* Tau = 0.383
Metenolone	7	Y	p < 0.001*	p < 0.0001** Tau = −0.490
Testosterone enanthate	6	Y	p = 0.015	p < 0.002* tau = −0.362

Significance level taken at P < 0.0025 with Bonferroni correction.

p≤0.0025 (*) p≤0.0005 (**) p≤0.00005 (***).

Statistically significant trends were identified in all the AAS except Boldenone. Anavar, Trenbolone, and Masteron had statistically significant upward trends. Stanazolol, Dianabol, Sustanon, Nandralone, Metenolone and Testosterone enanthate had statistically significant downward trends.

## Discussion

This study is the first to apply time series analysis to GT data for named AAS compounds in the UK. With Google having 86.7% of the market share for internet searches, and over 50,000 searches a second (Statista, 2016), the coverage provided by this study is broad in comparison with UK based surveys. The standout finding in this study is the presence of clear and consistent seasonal variation with 9 of the 10 compounds reaching statistical significance. Peaks of search interest, noted from April to July, may correspond to what Sassatelli (2010) describes as a ‘bulimic seasonality’ in fitness magazines, reflecting an interest in attaining an attractive physique in time for summer. This observed seasonality may support the notion of steroid use being part of health and beauty consumerism (Brennan et al., 2016), as opposed to being primarily related to body dysmorphia or a substance use disorder. Health promotion and prevention messages, as well as harm reduction services may be more effective if targeted during these periods of increased internet search interest. Similar recommendations have been made in response to seasonal spikes in sexually transmitted infections (Cornelisse, Chow, Chen, Bradshaw, & Fairley, 2015) with individuals preferring health messages when they are at increased risk. It is important to note however that harm reduction policies do not address the underlying neoliberal and consumerism driven risk normalisation underlying steroid use, which also needs to be addressed.

Our findings do need to be interpreted cautiously for a number of reasons. Firstly, it is not possible to know the motivation behind the Google searches. Compounds such as Trenbolone for example are used as a veterinarian steroid, and a portion of search interest may reflect this. It is notable however, that in the UK, Directive 96/22/EC severely restricts veterinarian AAS use (EU Monitor, 2004). Furthermore, with hundreds of thousands of websites focussing on unsanctioned human use, (Brennan, Kanayama & Pope, 2013), it is reasonable to believe that anabolic steroid web content is skewed towards image and performance enhancement.

Searches may be performed by researchers, journalists and out of curiosity and does not indicate intention to use or purchase. Furthermore, it is possible that a Google search for one AAS provides access to websites with information on the full range of compounds, reducing the need for further searches. This study did not use colloquial terms for various steroid products, rather names derived from an earlier published study (Cordaro et al., 2011). It is worth emphasising however that the use of colloquial terms notwithstanding, steroid users are known to thoroughly research their cycles (Andreasson & Johansson, 2015) and are likely to require the precision that comes from scientific names when performing web searches.

The lack of demographic data on who is performing the Google searches limits the inferences we can make. Trends may also be influenced by a number of confounding factors such as a general web interest in other issues or incidental news stories creating a spike in search interest in specific compounds. These limitations have been identified in other GT work for example in the prediction of influenza outbreaks (Eysenbach, 2011).

For the reasons above, interpretation of the ranking by RSV and the upward and downward trends is problematic. Nevertheless, it is notable that all the injectable steroids with the exception of Primibolan are represented in both this study and a 2015 AAS national survey (Bates & McVeigh, 2016), with Trenbolone being particularly prominent. Trenbolone was traditionally considered high risk and shunned by experienced bodybuilders in the 1980s (Monaghan, 2001) and its rise in internet search popularity may therefore warrant further investigation. Brennan et al. (2013) highlight Trenbolone as a particular concern, with over 4500 websites regarding its unsanctioned use, limited medical literature and a presumed low risk of criminal conviction due to its veterinarian legitimacy in some jurisdictions.

The large number of searches occurring for Dianabol and Anavar may have significance with regards to current health service provision. These are both oral compounds, potentially more hepatotoxic (Nieschlag & Vorona, 2015b), and may act as gateway drugs to injectable steroids (Hildebrandt, Harty & Langenbucher, 2012). With the main focus of current UK services for steroid users being NSPs (Hope et al., 2016), policy-makers may wish to evaluate to what extent the needs of oral steroid users are being met. It is worth noting that internet searches are far less likely to be met with sanctioned harm reduction or health promotion messages with such websites being substantially overwhelmed by pro-steroid content (Brennan et al., 2013).

The presence of fluctuating internet search volume of individual compounds, in the context of a perceived rise in AAS use overall, may be an area worth further investigation. While it is not possible to draw conclusions on how search interest relates to levels of use, time trend variation in levels of interest in different AAS may gives some indication of what is occurring within the wider AAS using community. More detailed web-analytics work on internet vendor sites could link web searches to purchases (Curtis et al., 2015). This is of particular interest if prospective users seem to show greater interest in substances which are associated with higher risks.

In conclusion, with current limited real-time, detailed AAS use surveillance, alternative data sources such GT may provide useful additional information. Cautious interpretation of GT data is recommended and future work involving triangulation with other data sources may make this method useful as a near real-time barometer of AAS interest, and variations over time.

## Conflict of interest statement

No conflict of interest declared.
