# Supplementary material for: Tracking internet interest in anabolic-androgenic steroids using Google Trends
Source: Int J Drug Policy. 2018 Jan;51:52–5. doi: 10.1016/j.drugpo.2017.11.001 (PMC5788329; doi:10.1016/j.drugpo.2017.11.001)
Supplement: Supplementary file 1 [file mmc1.docx]

**Table 1: Statistical tests for Google Trends data.**

| AAS | Relative search volume | Seasonality present  on decomposition | Mean Relative Search Volume Apr-Jul | Mean Relative Search Volume Sep-Dec | Wilcoxon signed rank tests (Sep-Dec/Apr-Jul) | Seasonal Mann  Kendall Tests |
| --- | --- | --- | --- | --- | --- | --- |
| More popular | | | | | | |
| Oxandralone  (Anavar) | 67 | Y | 80.15 | 53.05 | p<0.001 | p<0.001  Tau= +0.628 |
| Trenbolone Acetate  (Trenbolone) | 52 | Y | 57.80 | 45.15 | p<0.001 | p<0.001  Tau= +0.554 |
| Stanozolol  (Wnstrol) | 47 | Y | 66.00 | 57.00 | p<0.001 | p<0.001  Tau= -0.778 |
| Methandrostenolone  (Dianabol) | 45 | Y | 51.00 | 43.00 | P<0.001 | p=0.05  Tau= -0.144 |
| Sustanon | 23 | Y | 19.35 | 15.70 | P<0.001 | p<0.001  Tau= -0.907 |
| Less popular | | | | | | |
| Deca Durabolin  (Nandralone) | 11 | Y | 59.75 | 47.70 | p<0.001 | p<0.001  Tau= -0.756 |
| Boldenone undecylenate  (Equipoise) | 9 | Y | 51.20 | 43.0 | P<0.001 | p=0.25  Tau= +0.144 |
| Dromostanolone dipropoionate  (Masteron) | 9 | Y | 50.50 | 36.30 | p<0.001 | P<0.001  Tau= +0.383 |
| Metenolone enanthate  (Primibolan) | 7 | Y | 36.90 | 27.95 | p<0.001 | p<0.001  Tau= -0.490 |
| Testosterone enanthate | 6 | Y | 31.40 | 27.70 | p=0.014 | p<0.001  tau= -0.362 |

Significance level taken as ≤0.0025

**Figure 3: Decomposition graphs for the more popular AAS Jan 2011-Dec 2015**

| **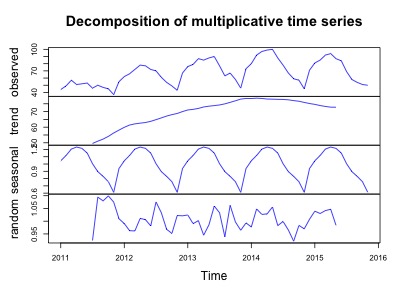** | **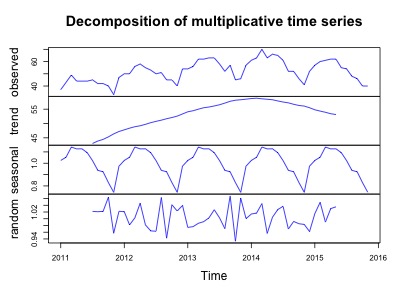** |
| --- | --- |
| **Anavar** | **Trenbolone** |
| **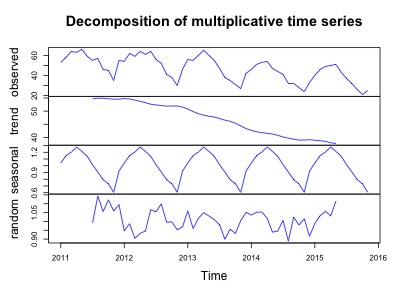** | 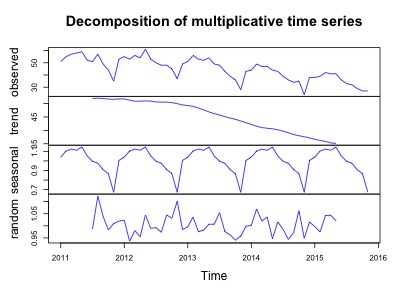 |
| **Stanozolol** | **Dianabol** |
| **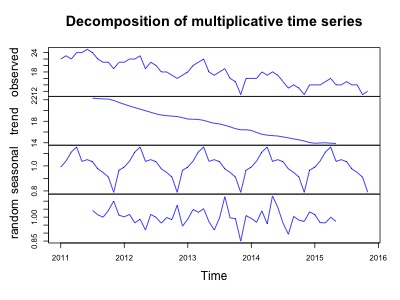** | |
| **Sustanon** | |

**Figure 4: Decomposition graphs for the less popular AAS Jan 2011-Dec 2015**

| **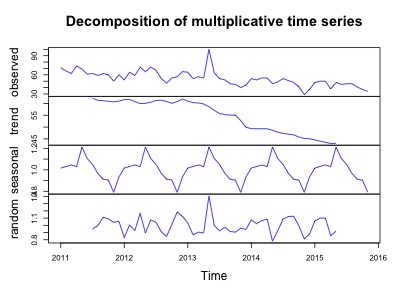** | **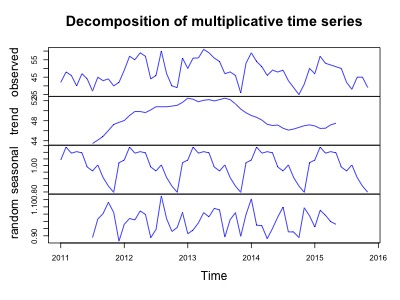** |
| --- | --- |
| **Nandralone** | **Boldenone** |
| **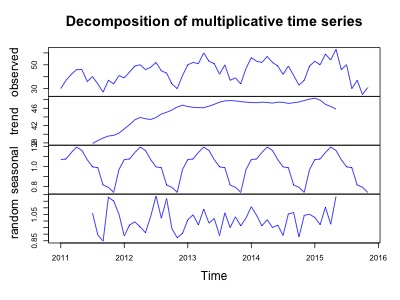** | 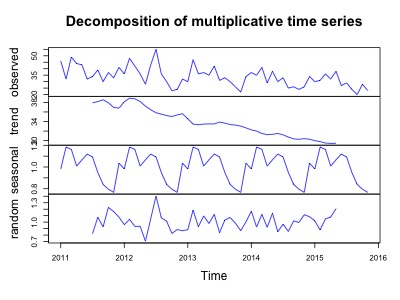 |
| **Masteron** | **Metenolone** |
| **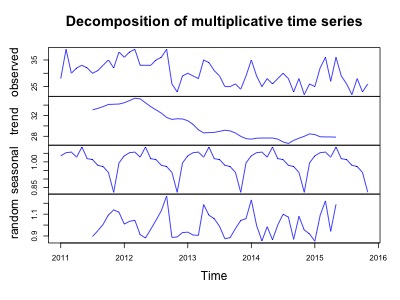** | |
| **Testosterone Enanthate** | |
